# Polysilicon-Channel Synaptic Transistors for Implementation of Short- and Long-Term Memory Characteristics

**DOI:** 10.3390/biomimetics8040368

**Published:** 2023-08-15

**Authors:** Myung-Hyun Baek, Hyungjin Kim

**Affiliations:** 1 Department of Electronic Engineering, Gangneung-Wonju National University, Gangneung 25457, Republic of Korea; mhbaek@gwnu.ac.kr; 2 Department of Electrical and Computer Engineering, Inha University, Incheon 22212, Republic of Korea

**Keywords:** neuromorphic computing, synaptic device, polysilicon, grain boundary, FN tunneling

## Abstract

The rapid progress of artificial neural networks (ANN) is largely attributed to the development of the rectified linear unit (ReLU) activation function. However, the implementation of software-based ANNs, such as convolutional neural networks (CNN), within the von Neumann architecture faces limitations due to its sequential processing mechanism. To overcome this challenge, research on hardware neuromorphic systems based on spiking neural networks (SNN) has gained significant interest. Artificial synapse, a crucial building block in these systems, has predominantly utilized resistive memory-based memristors. However, the two-terminal structure of memristors presents difficulties in processing feedback signals from the post-synaptic neuron, and without an additional rectifying device it is challenging to prevent sneak current paths. In this paper, we propose a four-terminal synaptic transistor with an asymmetric dual-gate structure as a solution to the limitations of two-terminal memristors. Similar to biological synapses, the proposed device multiplies the presynaptic input signal with stored synaptic weight information and transmits the result to the postsynaptic neuron. Weight modulation is explored through both hot carrier injection (HCI) and Fowler–Nordheim (FN) tunneling. Moreover, we investigate the incorporation of short-term memory properties by adopting polysilicon grain boundaries as temporary storage. It is anticipated that the devised synaptic devices, possessing both short-term and long-term memory characteristics, will enable the implementation of various novel ANN algorithms.

## 1. Introduction

The von Neumann computing system demonstrates superior performance to humans in processing tasks involving simple arithmetic. However, it is not suitable for cognitive tasks, such as pattern recognition, face detection, and natural language processing. In recent years, extensive research has been conducted on artificial neural network (ANN) algorithms that aim to mimic the multi-layered structure of the human brain [1]. The fundamental structure of these algorithms, known as “deep learning”, involves constructing networks that resemble the complex multilayered structure of the brain by mathematically modeling biological neurons. With advancements in various deep learning algorithms, artificial intelligence has achieved remarkable results in areas that exhibit more human-like characteristics.

**Figure 1:**
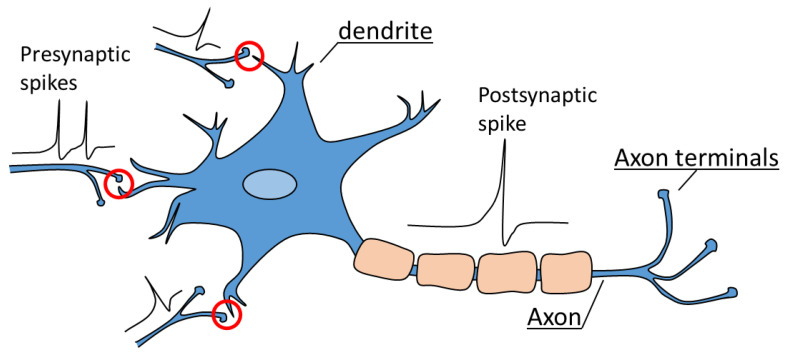
Schematic diagram of an actual neuron and synapse. A neuron is composed of a dendrite, an axon, and axon terminals. Red circle indicates synapses, which serve as connections between pre-neurons and post-neurons.

Deep neural network (DNN) algorithms are primarily grounded in mathematical models of neurons. To gain a deeper understanding, it is important to delve into the mechanisms of signal transmission within actual neurons. Figure 1 depicts a schematic diagram of a neuron and synapses in the nervous system. Synaptic transmission, also known as neurotransmission, refers to the transfer of information across biological synapses [2,3,4,5]. It plays a vital role in facilitating communication between two neurons, as the synapse connects the axon of a pre-synaptic neuron with the dendrite of a post-synaptic neuron. Action potentials, commonly referred to as spikes, are generated when the membrane potential of a neuron surpasses a threshold, and this potential is determined by the spatio–temporal integration of the neuron’s input signals. In other words, a post-synaptic neuron accumulates signals from multiple neurons connected through synapses over time, and when the total sum of these signals surpasses a certain threshold, it generates a signal to be transmitted to the next neuron. Consequently, the frequency and patterns of action potentials are influenced by synaptic transmissions. In this case, synapses do not simply transmit signals linearly. Even if the same spike occurs, some synapses can amplify the signal, while others can weaken it. This is known as synaptic plasticity. Similar to how humans can easily handle familiar tasks with minimal effort, synapses that receive repetitive stimulation gradually strengthen, enabling the generation of action potentials in the next neuron with just one or two spikes. On the other hand, synapses that receive very little stimulation weaken, resulting in a decrease in the strength of the signal transmitted to the next neuron. This is the reason why it is believed that memory and synaptic weights are closely related.

Hardware-based neuromorphic systems aim to implement deep neural networks (DNN) by emulating biological neurons and synapses [6]. Neurons are typically represented using CMOS circuits, while research on synapses is currently focused on various non-volatile memory technologies. An electronic neuron constructed with CMOS circuits possesses a functionality similar to the “integrate-and-fire (I&F)” mechanism of actual neurons. For instance, by utilizing capacitors, the incoming signals from various synapses can be integrated over time and, when the voltage of the capacitor surpasses a threshold, a spike can be generated.

Memristors, which based on two-terminal resistive memory, such as resistive random access memory (ReRAM) [7,8,9], phase change memory (PCM) [10,11], and spin-transfer-torque magnetic random access memory (STT-MRAM) [12], are regarded as adequate candidates as synapses due to their gradual switching phenomenon [13,14,15] and scalability to cross-point array [16,17,18,19,20]. Moreover, several pieces of research are focused on novel structures such as two-terminal fuse device [21], capacitor-based synapses [22], and optoelectronic synaptic devices [23]. However, it is difficult to process the feedback signal from the post-synaptic neuron, and there is a disadvantage that it is difficult to prevent the reverse current flow due to the absence of rectifying function. On the other hand, transistor-based synaptic devices are another candidate for artificial synapse [24,25]. Transistors can suppress current by controlling the gate voltage, making them immune to the sneak current issue. For storing synaptic weights, transistor-based synapses are primarily investigated with flash memory [26,27]. 

In this paper, the field-effect transistor (FET) based artificial synapse is investigated to compensate the demerits of memristor-based synapses. The proposed device features an asymmetric double gate structure and utilizes polysilicon as the channel material. Similar to charge trap flash (CTF), oxide/nitride/oxide triple layer is inserted at the bottom gate dielectric. Polysilicon channel can be easily fabricated using the chemical vapor deposition (CVD) method, making it convenient for the manufacturing process and suitable for three-dimensional stacking structures. Furthermore, the proposed device implements short-term memory characteristics by utilizing the grain boundaries of polysilicon, and it also simultaneously implements long-term memory characteristics using the storage nitride.

## 2. Materials and Methods

**Figure 2:**
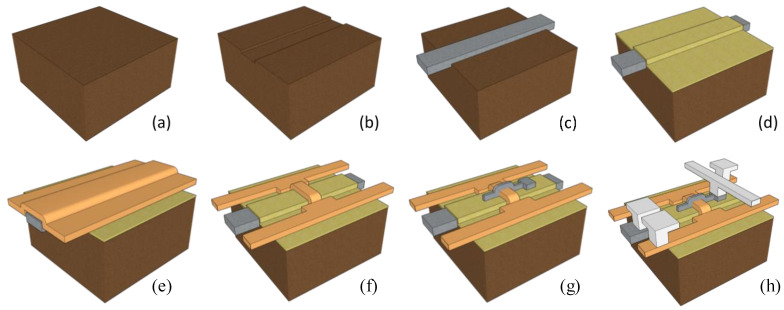
Brief fabrication methods for the proposed synaptic device. (**a**) BOX (buried oxide) oxidation, (**b**) BOX patterning for bottom gate line, (**c**) bottom gate formation, (**d**) oxide/nitride/oxide layer deposition, (**e**) active polysilicon layer deposition, (**f**) active layer patterning, (**g**) top gate formation, (**h**) metallization.

Figure 2 illustrates the fabrication process of the proposed synaptic transistor. First, a 400 nm buried oxide (BOX) was formed on a bare wafer using a wet oxidation process. The proposed synaptic device adopts a thin-film transistor (TFT) structure, allowing for easy isolation between each device through the BOX. Photolithography processes were performed to define the bottom gate lines, followed by the deposition of in-situ doped n+ polysilicon using LPCVD. The bottom gate lines were separated through a CMP process, and the bottom gate dielectric for long-term memory operation was deposited. The bottom gate dielectric consists of a 10 nm oxide/6 nm nitride/4 nm oxide structure, providing non-volatile memory functionality similar to that of CTF. A blocking oxide with a thickness of 10 nm was formed to prevent electron back tunneling phenomena that can occur during erase operations [28]. To further suppress back tunneling, materials with higher dielectric constants, such as Al_2_O_3_, can be used. For the tunnel oxide, a 4 nm SiO_2_ layer was applied to facilitate hot carrier injection (HCI) or Fowler–Nordheim (FN) tunneling. To construct the polysilicon body, amorphous silicon of 20-nm-thick was deposited using a 550 °C LPCVD process and crystallized by annealing at 600 °C for 24 h. After silicon recrystallization, patterning processes were performed to define the active region of the device. A 5 nm medium-temperature oxide (MTO) was deposited as the top gate oxide. Similar to the bottom gate, the top gate was deposited with in-situ-doped n+ polysilicon and patterned. An ion implantation process with 3 × 10^15^ cm^−2^ of As^+^ ions was carried out to form self-aligned source and drain regions. Finally, an interlayer dielectric (ILD) was deposited to protect the device, and the device was completed through contact hole etching and metallization. All device fabrication processes were conducted using the cleanroom facilities at the Inter-University Semiconductor Research Center (ISRC) at Seoul National University.

**Figure 3:**
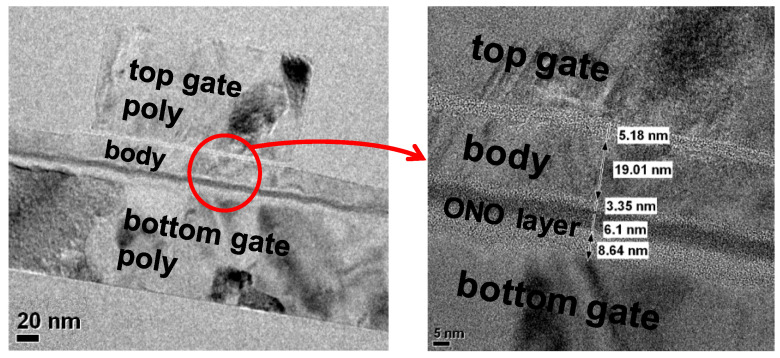
TEM image of fabricated device.

Figure 3 shows the TEM image of the fabricated synaptic transistor. The insulating layer between the bottom gate and the channel was formed as a CTF structure for storing synaptic weights. The blocking oxide is 8.6-nm thick to prevent electron back tunneling, while the tunnel oxide is designed to be 3.35-nm thick for efficient charge transfer. The top gate serves as the node for read operations (inference mode), and the gate oxide is formed to be 5-nm thick to achieve better gate controllability. Basic operation principles and Long-term memory property (synaptic weight) were measured with KEITHLEY 4200 SCS parameter analyzer. Short-term memory characteristics were investigated with a KEYSIGHT 4156C/B1500A semiconductor device parameter analyzer and B1530A Waveform Generator/Fast Measurement Unit (WGFMU). Temperature-dependency measurements were performed by applying proper heat to the bottom of the silicon wafer using a hot chuck installed on a probe station. Technology Computer Aided Design (TCAD) simulation was also performed using the SYNOPSYS Sentaurus device simulator. 

## 3. Results and Discussion

### 3.1. Basic Device Characteristics

**Figure 4:**
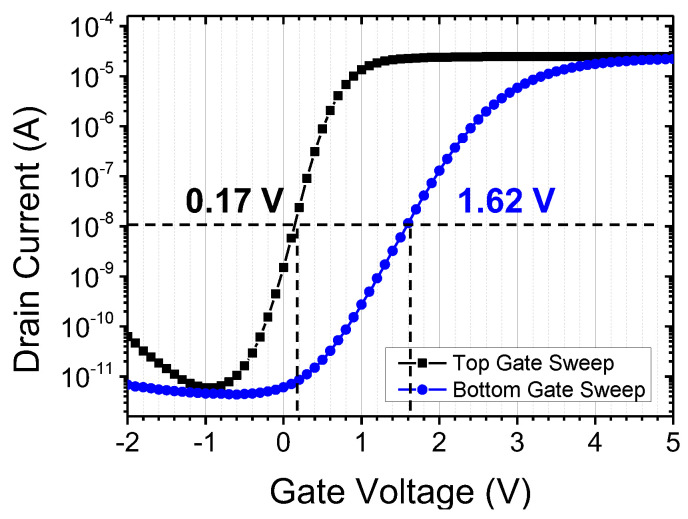
Transfer characteristics of top and bottom gate.

The proposed synaptic device in this study features an asymmetric double-gate structure. The bottom gate side is equipped with an Oxide/Nitride/Oxide layer for long-term memory, specifically for storing synaptic weights. The thick O/N/O layer inevitably weakens the channel control of the bottom gate. Therefore, when performing data read operations (verifying the stored synaptic weight information) using the bottom gate, the operating current may decrease. To compensate for this, the top gate adopts a single oxide layer with a thickness of 5 nm. In other words, the proposed device utilizes the bottom gate for synaptic weight updates and the top gate for transmitting signals during inference operations. Figure 4 represents the measurement results of the fabricated device. The channel length and width of the device are 1 μm and 20 μm, respectively. Constant drain voltage (V_d_) was applied with 0.5 V. When measuring the transfer characteristics of the top gate (the voltage applied to the top gate was swept from −2 V to 5 V in increments of 0.05 V), the voltage on the bottom gate is grounded and vice versa. The entire measurement process was conducted using the Keithley 4200 parameter analyzer [29]. According to Figure 4, it can be observed that the top gate and bottom gate independently influence the channel. The subthreshold slope (SS) of the bottom gate is larger due to the thicker memory node. The influence of channel control by the top gate and bottom gate can also be observed in the off-leakage region. During the top gate sweep, a significant occurrence of leakage current due to gate-induced drain leakage (GIDL) is observed below −1 V. In contrast, the bottom gate exhibits minimal GIDL. GIDL is a phenomenon where band-to-band tunneling occurs as a result of the vertical field at the drain side when a strong negative voltage is applied to the gate. The reduced occurrence of GIDL in the bottom gate can be attributed to the smaller field formed by the gate voltage in that region. The threshold voltage (V_t_) also shows a significant difference when measured with the top gate compared to the bottom gate. V_t_ was extracted where the drain current was 10 nA. As depicted in Figure 4, V_t_ of the top gate sweep and the bottom gate sweep is 0.17 V and 1.62 V, respectively. Due to the thick gate oxide stack and larger SS, the V_t_ on the bottom gate side is significantly higher than that on the top gate side. This can also affect the power consumption of the synaptic operation. The bottom gate requires a higher voltage to sufficiently turn on the channel, resulting in higher power consumption. Therefore, utilizing the top gate for the read operation of the synapse is more effective. In artificial neural network structures, synapses are typically ~10,000 times more abundant than neurons [30]. Thus, it is important to minimize the power consumption of the synapses as they account for a significant proportion of the overall system power. The proposed synaptic device also has the additional advantage of reducing power consumption by adopting polysilicon as the channel material. According to the measurement results, the operation current (transistor ‘on’ current) of the synapse is at the level of 30 μA/μm. While polysilicon has a lower electron mobility compared to single-crystalline silicon (at about 1/100), which could be a drawback for high-speed digital circuits, it acts as an advantage in synaptic applications in artificial neural networks.

**Figure 5:**
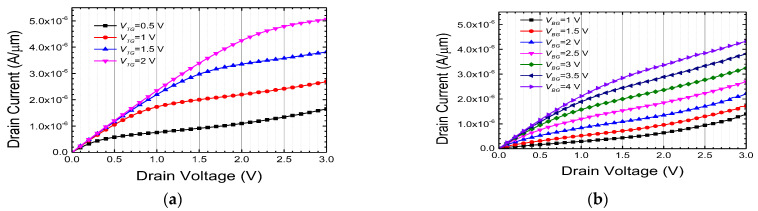
Output characteristics of (**a**) top gate and (**b**) bottom gate.

Output characteristics of each gate are also depicted in Figure 5 [29]. Output characteristics were measured by sweeping the drain voltage under various gate voltage conditions. It can be observed that the saturation current measured at the bottom gate is lower compared to the top gate, due to the thicker bottom gate dielectric with an O/N/O structure. At a reference current of 1 μA, there exists a voltage difference of approximately 2 V between the two gates, necessitating a 2 V higher voltage applied to the bottom gate in order to achieve the equivalent drain current. Notably, under high drain voltage conditions exceeding 2.5 V, the occurrence of impact ionization phenomena is more prominent in the bottom gate. This disparity can be attributed to the ineffective delivery of gate voltage to the channel by the O/N/O stack of the bottom gate, resulting in a larger lateral electric field between the channel and drain compared to when driven by the top gate. Consequently, the utilization of the bottom gate proves effective in inducing synaptic weight updates through the mechanism of HCI. Further elaboration on this topic will be provided in Section 3.3.

### 3.2. Grain Boundary Induced Short-Term Memory Effect

#### 3.2.1. Synaptic Plasticity

**Figure 6:**
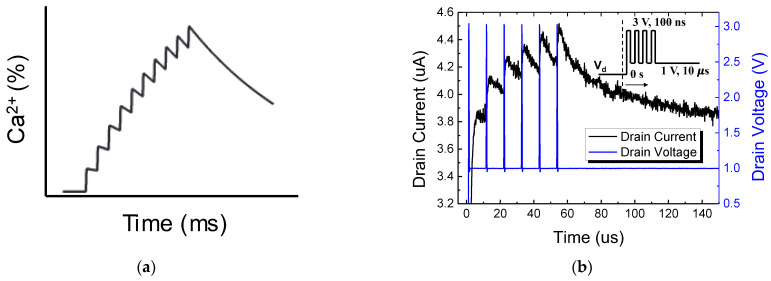
(**a**) Biological mechanisms of augmentation and post-tetanic potentiation (PTP). (**b**) Output response of fabricated synaptic transistor.

In the biological nervous system, the synaptic conductivity efficiency of the post-synaptic neuron, stimulated by the signals from the pre-synaptic neuron, exhibits flexibility rather than being fixed. For example, a synapse that receives continuous stimulation temporarily shows enhanced output for the same input signal. This phenomenon is similar to the temporary memory that occurs when a random sequence of numbers (such as a phone number) is repetitively recited. Post-tetanic potentiation is a prominent example of this, where the response to a short stimulus increases after sustained and repetitive stimulation [31,32,33]. An increase in calcium concentration at the synaptic terminal plays a crucial role, as it leads to an increase in neurotransmitter release from the synaptic vesicles. Figure 6a illustrates the occurrence of post-tetanic potentiation in biological synapses. 

In this study, we implemented the aforementioned biological post-tetanic potentiation behavior by adopting polysilicon as the channel material of the synapse device. Figure 6b demonstrates that, when serial short pulses are applied to the synapse (drain node), the drain current (output of the synapse) continues to increase, resembling the biological post-tetanic potentiation shown in Figure 6a. Unlike the actual nervous system where calcium ions are involved, artificial synapses operate with the movement of electrons, making their operational speed much faster. Once the sequence of pulses is terminated, the drain current gradually decreases, returning to its initial state. The short-term memory characteristics of the synaptic device can be interpreted as the trapping of charges at the grain boundaries within the polysilicon [34,35].

#### 3.2.2. Charge Trapping Phenomenon at Polysilicon Grain Boundary

**Figure 7:**
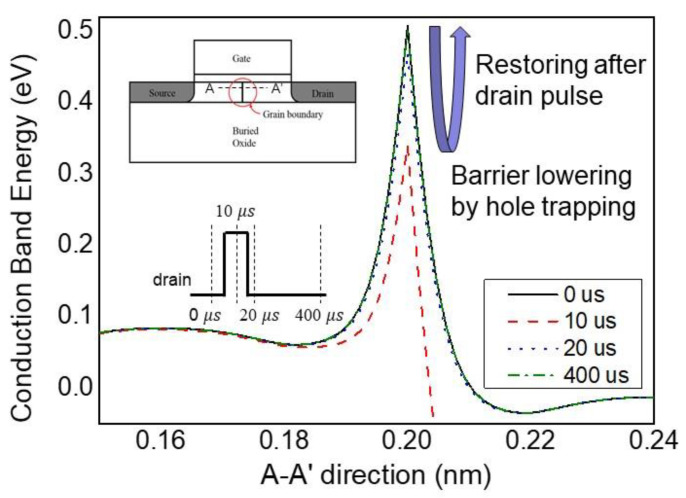
TCAD simulation of energy band structure under drain pulse response.

To validate this interpretation, TCAD simulations were performed. The simulation structure involved a polysilicon thin-film transistor (TFT) with identical vertical grain boundary inserted in the middle of the channel (Figure 7) [36,37]. In order to replicate the characteristics of polysilicon grain boundaries, interface traps were incorporated, and the trap density was assessed within the range of 10^13^–10^15^ cm^−3^ [38,39]. The results presented in Figure 7 illustrate the outcomes obtained with a trap density of 1 × 10^14^ cm^−3^. In order to emulate the fabricated device accurately, the body thickness was specified as 20 nm. To analyze the response of the drain current when pulses are applied to the drain, energy band diagrams were examined over the pulse duration. Prior to the pulse application, the grain boundary is occupied with channel electrons, forming a high-energy barrier (black line). On the other hand, when a high-voltage pulse capable of generating impact ionization at the drain side is applied, the generated holes are trapped at the grain boundary, temporarily lowering the electron barrier (red line). Based on the simulation results, it can be observed that the capture of excess holes reduces the energy barriers for electrons by approximately 0.15 eV. This reduction in energy barriers has the potential to cause a transient increase in the current flowing through the channel. After the pulse ends, the trapped holes recombine with electrons and gradually disappear, while the barrier recovers to its high state (blue & green line). Due to a certain time delay required for complete hole recombination, the application of a successive drain pulse to the device before the retention time results in the generation of additional holes and subsequent reduction of the barriers. The measurements presented in Figure 6b depict a scenario where drain pulses of 3 V and 10 ns are periodically applied at intervals of 10 µs. This observation suggests a progressive lowering of the barriers as the subsequent pulse is applied before the complete disappearance of all captured holes.

**Figure 8:**
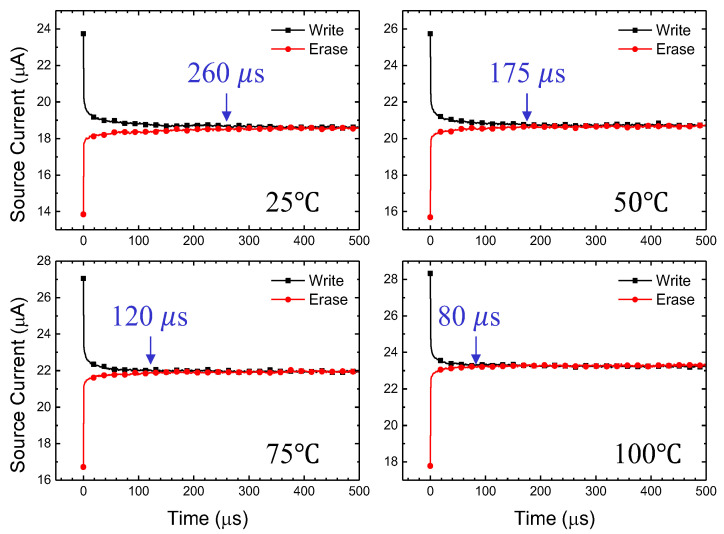
Temperature dependency for hole retention time.

Figure 8 shows the measurement results of hole retention time according to the temperature of the fabricated device. After the drain ‘write’ pulse (as illustrated at the inset of Figure 7), the holes generated by impact ionization are captured at the grain boundaries, exhibiting short-term memory, and gradually recombine with electrons to return to the initial state. At higher temperatures, the lifetime of holes decreases, resulting in shorter retention time [40,41]. According to reference [41], the ionization coefficient of silicon decreases with temperature under a horizontal electric field condition of 4 × 10^5^ V/cm. Additionally, the dark current of a diode at the same voltage also decreases with temperature, while the breakdown voltage increases. In other words, at high temperatures, the number of holes generated by impact ionization decreases, leading to a reduction in the hole lifetime and, consequently, a decrease in the retention time.

### 3.3. Long-Term Memory Property

#### 3.3.1. Synaptic Weight Update by Hot Carrier Injection

In a manner similar to flash memories, the synaptic output current of the proposed device can be modified by changing the trapped charge density. Two primary programming (PGM) methods are commonly used: HCI and FN tunneling. In this section, we focused on investigating the HCI mechanism for modulating the synaptic weight of the device. HCI, also known as channel hot electron (CHE) injection, is an electron injection method used in conventional NOR flash memories [42,43]. The NOR flash structure is well-suited for synapse arrays because the source current naturally represents the result of weighted sum operations.

**Figure 9:**
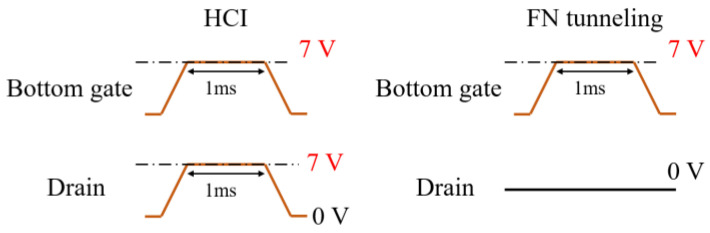
Pulse scheme for HCI and FN tunneling.

**Figure 10:**
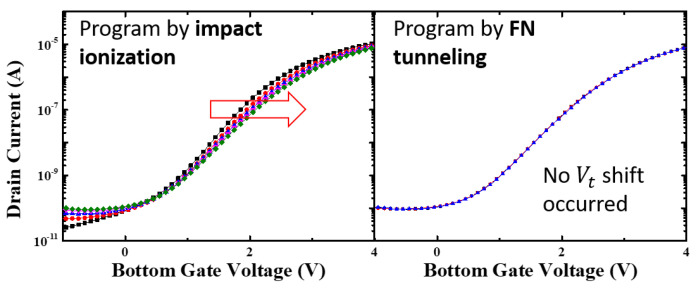
Measurement result for HCI and FN tunneling condition. Each line represents the altered transfer characteristics of the device with successive PGM pulse. Red arrow represents V_t_ shift due to trapped electrons.

**Figure 11:**
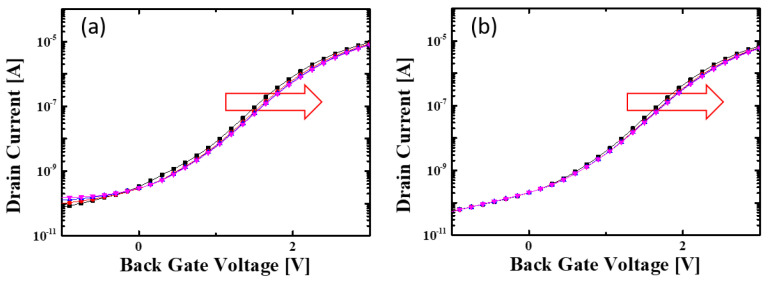
Charge injection by HCI when the bottom gate pulse magnitude is (**a**) 5 V, (**b**) 4 V respectively. Each line represents the altered transfer characteristics of the device with successive PGM pulse. Red arrow represents V_t_ shift due to trapped electrons.

The main distinction between HCI and FN tunneling lies in the magnitude of the drain voltage. HCI requires a high lateral electric field to generate “lucky” electrons capable of surpassing the oxide barrier, whereas FN tunneling relies on a strong vertical electric field to induce electron tunneling. To demonstrate the weight modulation effect using HCI, appropriate pulses were applied to the bottom gate and the drain. The bottom gate pulse has a magnitude of 7 V and a width of 1 ms to create a vertical injection field. Synchronized 7 V pulse trains are applied to the drain to accelerate the electrons, while the top gate and source nodes are grounded. A control group measurement is also conducted by applying 0 V to the drain to confirm that 7 V is insufficient for FN tunneling. The bias conditions for each scenario are presented in Figure 9. At this time, the top gate and source terminals are grounded [29]. The measurement results indicate that only the HCI condition leads to electron injection (Figure 10). Furthermore, Figure 11 demonstrates that the bottom gate bias can be reduced to 4 V. However, the injected electrons only cause a minimal change in the current induced by the bottom gate, which is not significant enough to reflect on the top gate. This is primarily due to the fact that the number of “lucky” electrons is essentially proportional to the current density of the device. Since the proposed synaptic transistor adopts polysilicon as the body material, which typically exhibits an electron mobility approximately one hundredth that of crystalline silicon, PGM through HCI is not suitable for polysilicon devices. In the next section, the FN tunneling mechanism will be evaluated as a charge injection principle to overcome the limitation imposed by the small current density.

#### 3.3.2. Synaptic Weight Update by Fowler–Nordheim Tunneling

**Figure 12:**
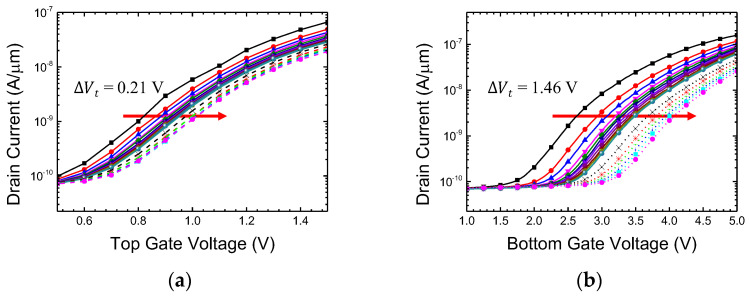
V_t_ shift by FN tunneling condition (PGM). Transfer characteristics for (**a**) top gate voltage and (**b**) bottom gate voltage. Each line represents the altered transfer characteristics of the device with successive PGM pulse.

Weight modulation measurements were conducted using the FN tunneling condition. The measured device had a top gate length (L_T_) of 300 nm. In Figure 12, the PGM operation, which can be interpreted as synaptic depression, was performed by applying a high positive bias to the bottom gate node [29,44]. Initially, 10 V pulses with a duration of 1 ms were applied to the bottom gate (solid lines), while the other nodes such as the source, drain, and top gate were grounded. The transfer characteristics of both the top and bottom gates exhibited a parallel threshold voltage (V_t_) shift, indicating the movement of electrons into the nitride layer. After several identical pulses, current modulation reached saturation due to the repulsive force of the trapped electrons. To assess the memory window, an additional 11 V pulse train was applied. As a result, a total voltage shift of 1.46 V was observed with respect to the bottom gate, while the ∆V_t_ for the top gate was 0.21 V. This difference in voltage shift is attributed to the polysilicon body thickness of approximately 19 nm in the fabricated device. To expand the memory window further, higher voltages can be applied to the bottom gate. In this experiment, PGM voltage was limited to 11 V, but in practical flash memory operation, gate voltages exceeding 15 V are utilized, enabling larger memory windows and a greater number of multi-state weight variations in the fabricated device. Moreover, in this study, pulses with uniform amplitudes were employed, inspired by the consistent size of spikes in biological synapses. However, in actual flash memory, the PGM operation adopts the incremental step pulse programming (ISPP) method, which leads to a more linear threshold voltage variation in response to the applied pulses [44,45,46]. While complete replication of biological synaptic behavior is not the goal, incorporating the ISPP method for synaptic weight modification could potentially yield more efficient outcomes.

**Figure 13:**
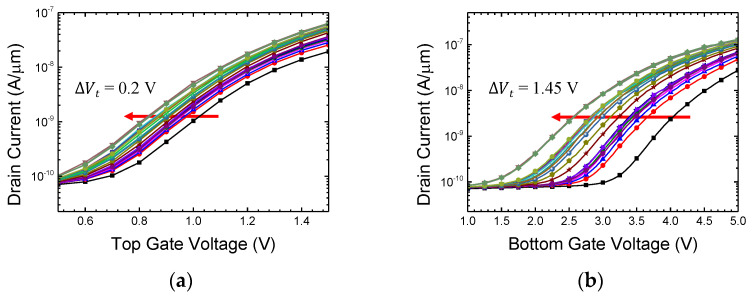
V_t_ shift by FN tunneling condition (ERS). Transfer characteristics for (**a**) top gate voltage and (**b**) bottom gate voltage. Each line represents the altered transfer characteristics of the device with successive PGM pulse.

Next, the erase (ERS) operation verification was performed. In contrast to the PGM operation, −10 V pulses were applied to the bottom gate. Since the fabricated device had a planar structure and the blocking oxide was formed by SiO_2_, there was a risk of electron back tunneling if the negative voltage bias was too large [47,48]. Therefore, various pulse widths ranging from 1 ms to 100 ms were investigated to achieve a specific amount of ERS window with low electric field (−10 V). As a result, a negative shift similar to that observed in the PGM measurement was obtained (Figure 13). That is, it can be confirmed that all the electrons injected during the PGM operation had been expelled. Furthermore, in order to widen the potentiation window through hole injection, suppression of back tunneling during the ERS operation is necessary. This can be achieved by applying high-k materials such as Al_2_O_3_ to the blocking oxide, or structural advances such as 3-D cylindrical (macaroni) structure [49,50].

**Figure 14:**
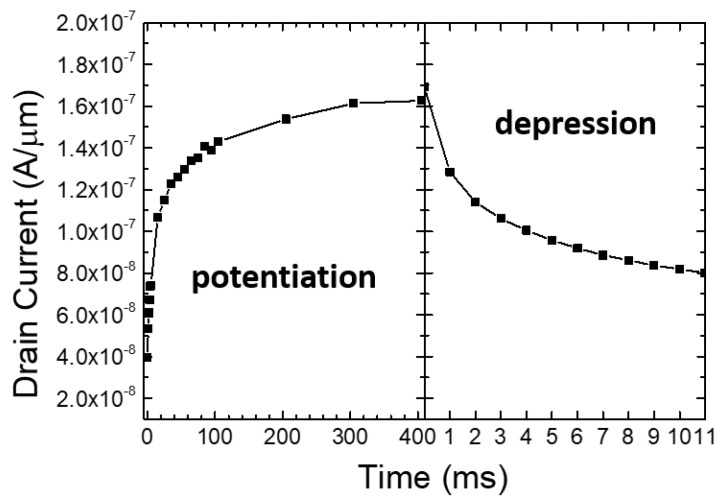
Synaptic weight representation (potentiation & depression).

To derive synaptic weights from the PGM/ERS data (Figure 12 and Figure 13), the current value under specific read operation conditions was extracted. The read operation was performed with V_t,read_ = 2 V and V_D_ = 1 V. Figure 14 illustrates the range of weight values represented by the source current. The data points on the graph represent the changes in drain current corresponding to the applied pulses, thereby indicating the total number of points as a representation of the multilevel operation of synaptic weights. This demonstrates the device’s ability to achieve multilevel capability in both synaptic potentiation (ERS) and depression (PGM) cases. 

**Figure 15:**
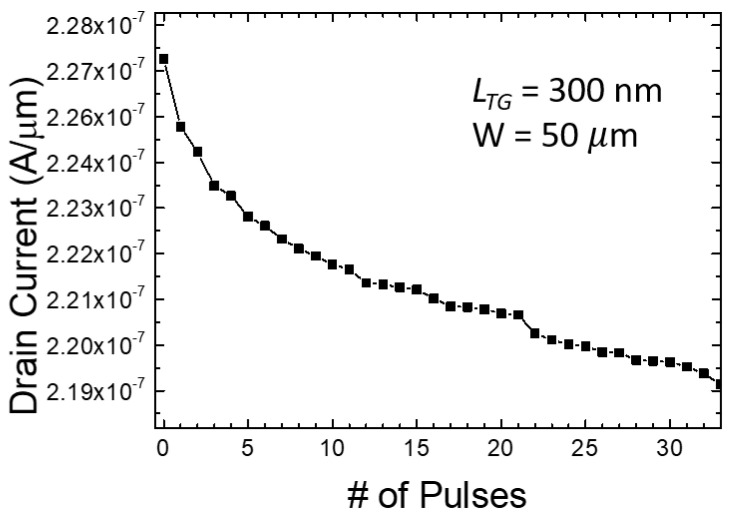
Multilevel weight representation more than 5 bits by consecutive 10 V pulses.

To achieve a higher number of multi-level implementations, read operations were performed with 33 consecutive 10 V, 10 µs pulses applied to the bottom gate, and a read operation were performed between each PGM pulses. The measurement result indicates that the fabricated synaptic transistor can achieve more than 5 bits of distinguishable level (Figure 15). As previously mentioned, in the case of potentiation, enhancing the ERS voltage along with the blocking oxide can lead to improvements in ERS time and an increased number of achievable multilevel states. Similarly, for depression, the adoption of techniques such as ISPP allows for the implementation of a greater variety of multilevel operations.

### 3.4. Performance Benchmark

In synaptic devices, important factors include the size of the device, memory density, operating voltage, power consumption, On/Off ratio (sneak current), operational speed, and the ability to achieve multi-level conductance states. Since synapses are over ~10,000 times more numerous than neurons, memory integration density is crucial, and power consumption should also be minimized. Additionally, as the learning accuracy increases with finer weight adjustments, the ability to achieve multi-level weight control is important. Table 1 presents a performance benchmark of various types of synaptic devices. Compared to memristor synapses such as ReRAM and PCM, the proposed polysilicon-based double-gate synaptic device exhibits an on/off ratio exceeding 10^6^. This high on/off ratio allows for perfect implementation of the off state, completely suppressing the sneak current issue. However, in terms of area efficiency, the proposed synaptic transistor requires approximately 2.5 times more area, with a 10F^2^ structure, compared to the typical 4F^2^ structure of memristor-based devices. Furthermore, for future improvement studies, it is necessary to assess the feasibility of achieving more multi-level weight states by applying smaller set pulses.

## 4. Conclusions

We propose a polysilicon TFT-based artificial synaptic device for emulation of biological synapses. The proposed device was fully fabricated by CMOS technology at ISRC, Seoul National University. Biological synapses exhibit synaptic plasticity, where the strength of synaptic connections varies based on the intensity of stimulation. The fabricated device exhibits short-term memory characteristics similar to those of synaptic plasticity, which is attributed to the grain boundary effect in the polysilicon channel. The TCAD simulation results support the mechanism of short-term memory effect caused by grain boundaries. We design the device to store synaptic weights by utilizing nitride as a long-term memory node, similar to CTF. We compare and measure the modulation of synaptic weights using HCI and FN tunneling methods for long-term memory. The measurement results show that the FN tunneling method is much more efficient in obtaining weight update efficiency. Based on the experimental results of non-volatile memory operation, the capability of the fabricated synapses to exhibit multi-level weight variations was demonstrated. The synaptic weights could be adjusted to a total of 33 distinct levels, and further refinement can be achieved by increasing the operating voltage or employing the ISPP method. We expect that the proposed device will serve as a foundation for implementing low-power neuromorphic systems.

## Figures and Tables

**Table 1:** Benchmark for various synaptic devices.

**Device Type**	ReRAM (ETML/HfO_x_) [51]	PCM (GST) [52]	Polysilicon Flash (ONO) [26]	This Work
R_ON_	100 KΩ	40 KΩ	530 MΩ	90 KΩ
On/Off ratio	10	12.5	62.2	~10^6^
Number of multi-level weight states	7 bit	10 bit	4 bit	5 bit
Area	21.17 mm^2^	22.18 mm^2^	33.27 mm^2^	55.45 mm^2^
Operating voltage	1.6 V	~2 V	3 V	1 V
Power consumption	25.6 μW	100 μW	16.8 nW	11.4 μW
SET pulse	50 ns	100 ns	100 μs	1 ms

## Data Availability

Not applicable.
